# Maturation of the Mfa1 Fimbriae in the Oral Pathogen *Porphyromonas gingivalis*

**DOI:** 10.3389/fcimb.2018.00137

**Published:** 2018-05-09

**Authors:** Jae Y. Lee, Daniel P. Miller, Leng Wu, Carolyn R. Casella, Yoshiaki Hasegawa, Richard J. Lamont

**Affiliations:** ^1^Department of Oral Immunology and Infectious Diseases, University of Louisville, Louisville, KY, United States; ^2^Department of Microbiology and Immunology, University of Louisville, Louisville, KY, United States; ^3^Department of Microbiology, School of Dentistry, Aichi Gakuin University, Nagoya, Japan

**Keywords:** donor strand exchange, polymerization, hydrophobicity, accessory fimbrial proteins, periodontal disease

## Abstract

The Mfa1 fimbriae of the periodontal pathogen *Porphyromonas gingivalis* are involved in adhesion, including binding to synergistic species in oral biofilms. Mfa1 fimbriae are comprised of 5 proteins: the structural component Mfa1, the anchor Mfa2, and Mfa3-5 which constitute the fimbrial tip complex. Interactions among the Mfa proteins and the polymerization mechanism for Mfa1 are poorly understood. Here we show that Mfa3 can bind to Mfa1, 2, 4, and 5 *in vitro*, and may function as an adaptor protein interlinking other fimbrial subunits. Polymerization of Mfa1 is independent of Mfa3-5 and requires proteolytic processing mediated by the RgpA/B arginine gingipains of *P. gingivalis*. Both the N- and C- terminal regions of Mfa1 are necessary for polymerization; however, potential β-strand disrupting amino acid substitutions in these regions do not impair Mfa1 polymerization. In contrast, substitution of hydrophobic amino acids with charged residues in either the N- or C- terminal domains yielded Mfa1 proteins that failed to polymerize. Collectively, these results indicate that Mfa3 serves as an adaptor protein between Mfa1 and other accessory fimbrial proteins. Mfa1 fimbrial polymerization is dependent on hydrophobicity in both the N- and C-terminal regions, indicative of an assembly mechanism involving the terminal regions forming a hydrophobic binding interface between Mfa1 subunits.

## Introduction

*Porphyromonas gingivalis*, a Gram-negative anaerobe, is a major etiological agent in periodontal disease, one of the most common infectious diseases of humans (Lamont and Jenkinson, 1998; Kassebaum et al., 2014; Hajishengallis, 2015; Tonetti et al., 2017). Periodontitis is a microbial community disease, and *P*. *gingivalis* is considered a keystone pathogen in that it can exert a disproportional influence on nososymbiocity, the pathogenic potential of the polymicrobial community (Hajishengallis et al., 2012; Hajishengallis and Lamont, 2016). *P. gingivalis* is well equipped with virulence factors to facilitate colonization and induction of dysbiotic inflammatory responses (Lamont and Jenkinson, 1998; Hajishengallis and Lamont, 2014; Lamont and Hajishengallis, 2015). Moreover, *P. gingivalis* engages in an interactive molecular dialog with other community participants, utilizing both physical and chemical communication (Wright et al., 2013; Kuboniwa et al., 2017).

*P. gingivalis* cells possess at least two types of fimbrial structure which mediate attachment to the hard and soft tissues of the oral cavity (Yoshimura et al., 2009; Enersen et al., 2013). The major, or long, fimbriae comprise the FimA structural subunit protein and extend up to 3 μm from the cell surface (Enersen et al., 2013). FimA has been shown to interact with β1-intgerin surface proteins of gingival epithelial cells, an event that can stimulate subsequent internalization of the organism (Yilmaz et al., 2002). FimA also binds to GAPDH on the surface of oral streptococci, which are primary colonizers of the oral cavity and provide attachment sites for later-colonizing bacteria such as *P. gingivalis* (Maeda et al., 2004; Sakanaka et al., 2016). In addition, contact between *Streptococcus gordonii* and *P. gingivalis* raises the pathogenic potential of the dual species community (Daep et al., 2011). The shorter or minor fimbriae comprise the Mfa1 structural subunit protein and range in length from 60 to 500 nm (Hamada et al., 1996; Park et al., 2005). Mfa1 fimbriae bind to the SspA/B surface protein of *S. gordonii*, and this binding initiates a signal transduction event within *P. gingivalis* that is based on protein tyrosine (de)phosphorylation (Maeda et al., 2008; Wright et al., 2013, 2014). Another reported binding partner for Mfa1 fimbriae is the DC-SIGN (dendritic cell-specific ICAM-3 grabbing nonintegrin) receptor of human dendritic cells. Interaction between Mfa1 and DC-SIGN facilitates the entry of *P. gingivalis* into dendritic cells and subsequent persistence of *P. gingivalis* within the cells, leading to blocked maturation of dendritic cells, stimulation of Th2 effector response, and diminished levels of pro-inflammatory cytokines (Zeituni et al., 2009; El-Awady et al., 2015; Arjunan et al., 2016).

In addition to the Mfa1 structural protein, mature Mfa1 fimbriae contain accessory proteins Mfa2-5 (Hasegawa et al., 2016). Transcriptional analysis shows that *mfa1, mfa2, mfa3*, and *mfa4* are arranged in an operon while *mfa5* is transcribed independently (Hasegawa et al., 2009). Mfa2 is located in the basal portion of the fimbriae and functions as an anchor as well as an assembly and elongation terminator (Hasegawa et al., 2009). Mfa3, Mfa4, and Mfa5 all participate in the assembly of an accessory protein supramolecular complex, and the absence of any one of the three proteins results in fimbriae that lack all three accessory fimbrial proteins (Hasegawa et al., 2013, 2016; Ikai et al., 2015). Mfa3 is localized in the distal tip portion of the Mfa1 fimbriae, and thus may play a role as a ligand to receptors on host cells and other oral bacteria (Hasegawa et al., 2013). Processing and surface expression of Mfa1, Mfa3, and Mfa4 occurs first by signal peptidase II, and the subsequent lipoprotein is trafficked to the cell surface where it is cleaved by Rgp to yield the mature form (Shoji et al., 2004; Ikai et al., 2015). Mfa5, which contains a von Willebrand factor type A domain, is translocated to the cell surface by the Type IX Secretion System (T9SS) (Hasegawa et al., 2016).

One well-characterized fimbrial polymerization mechanism is donor-strand exchange (DSE) (Zav'yalov et al., 2010). In the type 1 and P pili systems of *E. coli*, for example, the fimbrial proteins possess a C-terminal conserved, incomplete IgG fold that lacks a β-strand (Waksman and Hultgren, 2009). This fold creates a hydrophobic groove which interacts with an N-terminal β-strand (NTD) present on another subunit, thus forming a dimeric structure (Waksman and Hultgren, 2009; Hospenthal et al., 2017). Processive interactions between two termini of nascent subunits yields the polymeric backbone of the fimbrial structure (Waksman and Hultgren, 2009; Hospenthal et al., 2017). The crystal structures of the FimA and Mfa1, along with mechanistic studies, indicate that *P. gingivalis* utilizes a DSE mechanism for fimbrial polymerization and biogenesis (Xu et al., 2016; Hall et al., 2018), although it is unclear whether the NTD or C-terminal β-strand (CTD) functions as the donor strand (Xu et al., 2016; Hall et al., 2018). In the current study, we embarked upon a more extensive analysis of the mechanism of Mfa1 polymerization and the interaction among Mfa accessory proteins.

## Methods

### Bacterial strains and growth conditions

Bacterial strains are listed in Table 1. *P*. *gingivalis* was cultivated anaerobically at 37°C in trypticase soy broth supplemented with 1 mg/ml yeast extract, 5 μg/ml hemin, and 1 μg/ml menadione. When appropriate, media were supplemented with 10 μg/ml erythromycin, or 1 μg/ml tetracycline. *Escherichia coli* was grown in Luria-Bertani media supplemented as needed with 100 μg/ml ampicillin, 50 μg/ml kanamycin.

**Table 1:** Bacterial strains used in this study.

**Bacteria/strain**	**Comment**	**Source**
***P. gingivalis***		
ATCC 33277	wild-type, Gm^r^	ATCC
Δ*mfa1*	*mfa1*-deletion mutant of 33277, Em^r^	This study
Δ*mfa2*	*mfa2*-deletion mutant of 33277, Em^r^	This study
Δ*mfa3*	*mfa3*-deletion mutant of 33277, Em^r^	This study
Δ*mfa4*	*mfa4*-deletion mutant of 33277, Em^r^	This study
Δ*mfa5*	*mfa5*-deletion mutant of 33277, Em^r^	This study
*ΔrgpA/B*	double deletion of *rgpA* and *rgpB* in 33277, Em^r^ Tc^r^	This study
*Δkgp*	*kgp* deletion of 33277 Em^r^	This study
CΔ*mfa1*	Δ*mfa1* complemented with *mfa1* in pT-COW Em^r^ Tc^r^	This study
NAlaΔ*mfa1*	Δ*mfa1* complemented with pT-COW containing *mfa1* with point mutations I73A, V76A, I78A, V81A, Em^r^ Tc^r^	This study
CAlaΔ*mfa1*	Δ*mfa1* complemented with pT-COW containing *mfa1* with point mutations V547A, V549A, V551A, V547A, Em^r^ Tc^r^	This study
NAspΔ*mfa1*	Δ*mfa1* complemented with pT-COW containing *mfa1* with point mutations I73D, V76D, I78D, V81D, Em^r^ Tc^r^	This study
CAsp Δ*mfa1*	Δ*mfa1* complemented with pT-COW containing *mfa1* with point mutations V547D, V549D, V551D, V547D, Em^r^ Tc^r^	This study
***Escherichia coli***		
BL21 Star (DE3)	Recombinant protein expression strain (inducible by IPTG via *lacUV* promoter and T7 polymerase)	Invitrogen

### Construction of gene-deficient and complemented strains

Allelic exchange mutants were generated by the PCR fusion technique (Simionato et al., 2006), using the primers listed in Table S1. Constructs were introduced into *P. gingivalis* by electroporation and the correct insertion confirmed by PCR and sequencing. For complementation, the gene coding region (full length or truncation derivatives) and *mfa1* promoter region were amplified using the primers listed in Table S1 and cloned into the shuttle vector pT-COW (Gardner et al., 1996). The resulting plasmid was introduced into the Δ*mfa1* mutant by electroporation. Complementation was confirmed by plasmid purification, sequencing and RT-PCR for gene expression. Site-specific mutations were introduced into *mfa1* using the Q5 Site-Directed Mutagenesis kit (New England Biolabs) with primers listed in Table S1. The construct was confirmed by sequencing, cloned into pT-COW and electroporated into Δ*mfa1*.

### Expression and purification of recombinant proteins

Proteins were either expressed as hexahistidine (His)-tagged or glutathione-S-transferase (GST)-tagged and purified from BL21 Star. His-tagged recombinant proteins were purified by immobilized metal ion chromatography using a nickel-charged resin. GST-tagged protein was purified through affinity chromatography using glutathione agarose resin (Genscript). The GST-tag was removed with PreScission Protease (Genscript). For purification of mature Rgp-processed Mfa4, purified His-tag RgpB (Veillard et al., 2013) was reacted with the Mfa4 precursor protein overnight at room temperature. The cleaved N-terminal segment and His-tagged RgpB were separated by Ni-chromatography. Proteolytic cleavage was confirmed by SDS-PAGE.

### Antisera

Rabbit polyclonal antisera to recombinant Mfa2 and Mfa3 were generated by Abgent (San Diego, CA). Rabbit polyclonal antiserum to recombinant Mfa4 and Mfa5 was generated by Enzymax (Lexington, KY). Mfa1 antibody has been described previously (Park et al., 2005).

### ELISA

Recombinant Mfa proteins or BSA (1 μg each) were immobilized on a 96-well microplate (Corning). Wells were washed with PBS containing 0.1% Tween20 (PBST), and blocked with 10% skim milk in PBS for 1 h at room temperature. Potential binding partner recombinant proteins were added for 1 h at room temperature. Wells were washed twice with PBST, and primary antibodies diluted in 1% skim milk in PBST were added for 1 h at room temperature. Wells were washed again and reacted with horseradish peroxidase-linked secondary antibodies (Cell Signaling Technology) diluted 1:5,000 in 1% skim milk in PBST for 1 h at room temperature. After further washing, 3,3,5,5-tetramethylbenzidine (TMB; Thermo Fisher) substrate solution was added for 10 min at room temperature. The reaction was stopped with 100 μl 0.16 M sulfuric acid, and absorbance measured at 450 nm on a Victor X3 plate reader. The binding data were analyzed using a 1:1 saturation binding model (Graphpad Prism 6.04), the binding constants determined, and the fit of the data to the non-linear regression model is reported as *R*^2^.

### Reverse-transcriptase PCR

Total RNA was isolated from bacterial cultures at OD_600_ 0.5 using RNeasy (Qiagen). RNA was converted to cDNA with a High-Capacity cDNA Reverse Transcription Kit (ThermoFisher). Primers used are listed in Table S1. After amplification, PCR products were examined by agarose gel electrophoresis and staining with SYBR Safe (Invitrogen).

### Polymerization

Spontaneous polymerization of purified recombinant Mfa1 proteins was assayed by Western blotting. Mfa1 proteins (0.1 μg) were diluted in SDS-PAGE sample buffer and incubated at either 60° or 100°C for 10 min, fractionated by SDS-PAGE, electroblotted to nitrocellulose membranes and identified with anti-Mfa1 antisera (1:10,000) as described below.

### SDS-PAGE and immunoblotting

Proteins were separated by 10% SDS-polyacrylamide gel electrophoresis, blotted onto a nitrocellulose membrane and blocked with 10% skim milk in PBS containing 0.1% Tween20. Blots were reacted for 16 h with primary antibody at 4 °C followed by 1 h with HRP-conjugated secondary antibody at room temperature. The membrane was developed using ECL detection, and densitometric analyses conducted using a ChemiDoc XRS Plus (Bio-Rad).

## Results

### Binding interactions among Mfa proteins

**Figure 1:**
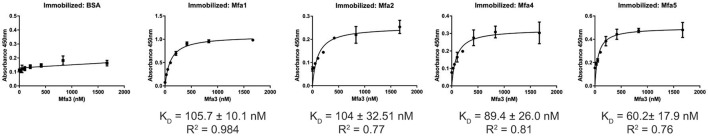
ELISA of binding between post-Rgp processed Mfa3 and other Mfa proteins. Rgp-processed forms of Mfa1 or Mfa4, along with recombinant Mfa2 or Mfa5 (1 μg) were immobilized on a microtiter plate. BSA was used as a control. Binding of Rgp-processed Mfa3 at the concentrations indicated was detected with antibodies to Mfa3 (1:5,000) followed by secondary anti-rabbit IgG HRP-linked antibodies (1:5,000). Signals were developed with the TMB substrate and absorbance values were measured at 450 nm. The binding data were analyzed using a 1:1 saturation binding model, and K_D_ and *R*^2^ values are shown. The data are from 3 independent experiments.

**Figure 2:**
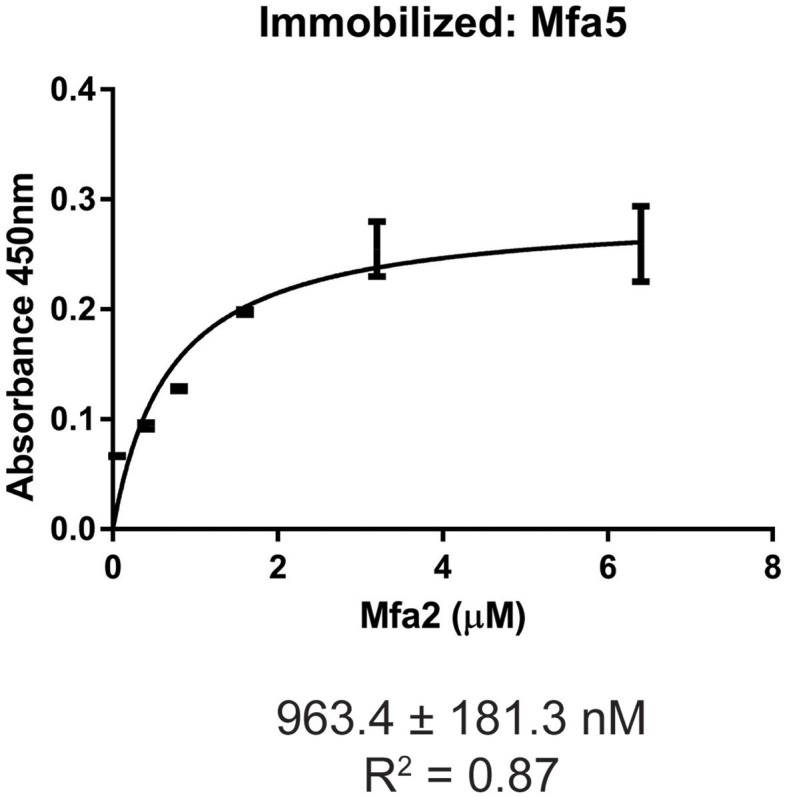
ELISA of binding between Mfa2 and Mfa5. Mfa5 (1 μg) was immobilized on a microtiter plate. Binding of recombinant Mfa2 at the concentrations indicated was detected with antibodies to Mfa2 (1:5,000) followed by secondary anti-rabbit IgG HRP-linked antibodies (1:5,000). Signals were developed with the TMB substrate and absorbance values were measured at 450 nm. The binding data were analyzed using a 1:1 saturation binding model, and K_D_ and *R*^2^ values are shown. The data are from 3 independent experiments.

The Mfa1 fimbrial structure of *P. gingivalis* is anchored by Mfa2 in the outer membrane. Mfa3-5, in contrast, assemble on the tip of the fimbriae, and loss of any one of these proteins results in the absence of all of them from the fimbriae (Hasegawa et al., 2009, 2013; Ikai et al., 2015). While Mfa1 and Mfa2 have been found to co-precipitate in whole cells (Hasegawa et al., 2009), physical interactions among the Mfa proteins have not been investigated in a systematic manner. Mature Mfa1, 3 and 4 proteins corresponding to the gingipain-processed forms were generated, and soluble Mfa5 protein was obtained by truncating the C-terminal region at aspartic acid residue 1044. As Mfa5 is secreted through the T9SS and has the C-terminus cleaved by PorU (Hasegawa et al., 2016), the functional consequence of such truncation is lessened. Mfa2 was produced as a full-length protein which is functional in other studies (Hasegawa et al., 2009). Although lipidation was not tested in this study, other reports have found that *P. gingivalis* proteins are lipidated in *E. coli* (Shoji et al., 2010). Binding among the Mfa proteins was measured using an ELISA, while recognizing the inherent limitations of this model including presentation of one of the binding partners deposited on a solid surface. As shown in Figure 1, the Mfa3 protein exhibited concentration-dependent binding activity to Mfa1, Mfa4, and Mfa5, indicating that Mfa3 may function as a bridging molecule interlinking Mfa1 and Mfa4-5. The affinity of Mfa3 binding to other fimbrial components was in the nanomolar range (Figure 1). In *E. coli*, the FimC usher protein has been shown to interact with other fimbrial structural and tip proteins (FimA, FimG, and FimF) with binding constants ranging from 176 to 1370 nm (Busch and Waksman, 2012). Interactions of the FimD chaperone with other fimbrial proteins display slightly weaker binding affinities between 1.2 to 3.4 μM (Nishiyama et al., 2003). Similarly, the *E. coli* PapC usher interacts with PapDG and PapDA with affinities ranging from 60 nm to 1.5 μM (Saulino et al., 1998). More accurate values have been obtained in the F1 capsular antigen system: binding between the Caf1M periplasmic chaperone and Caf1 pilus subunit is characterized by a Kd of 62.5 nM (Yu et al., 2012), binding between the Caf1M-Caf1 chaperone-subunit complex and the Caf1A usher is characterized by a Kd of 2420 nM (Di Yu et al., 2012), and binding between subunits in the Caf1 fiber is suggested to be extremely tight with a Kd > 10^−14^ M (Zavialov et al., 2005). In support of the concept that Mfa3 functions as an adaptor to integrate Mfa4 and Mfa5 into the fimbrial structure, Mfa1 did not bind to Mfa4 or Mfa5, and Mfa4 and Mfa5 did not bind to each other (Figure S1). Interestingly, Mfa2 also bound to Mfa3 (Figure 1) and Mfa5 (Figure 2), although the interaction with Mfa5 was in the micromolar range. Mfa2 did not bind to Mfa4 (not shown). Should Mfa2 binding to Mfa3 and Mfa5 occur *in vivo*, this may indicate a role for Mfa2 in regulation of the tip complex, and this notion is supported by reports that Mfa3 and Mfa4 can polymerize through DSE (Kloppsteck et al., 2016) and thus Mfa2 may limit extension of the tip complex.

### Role of accessory proteins in Mfa1 polymerization

**Figure 3:**
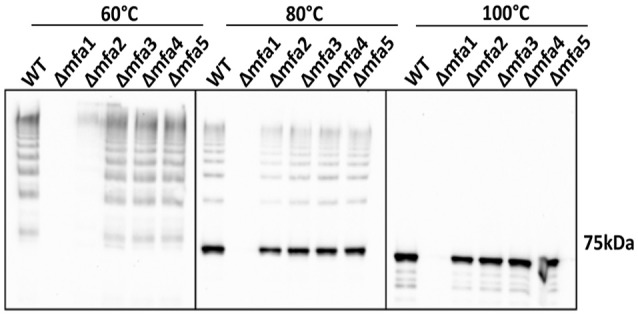
Mfa1 polymerization in *mfa* mutants. Whole cell lysates of 33277 WT and *mfa* mutants were treated at 60°, 80°, and 100°C prior to SDS-PAGE and immunoblotting with antibodies to Mfa1. Results are representative of 4 biological replicates.

To investigate the influence of Mfa accessory proteins on Mfa1 polymerization, we constructed isogenic mutations in each of the *mfa* genes. RT-PCR showed that deletion of one of the genes on the *mfa1*-5 cluster did not affect transcription of the other genes (Figure S2). Cell lysates were heated to different temperatures and immunoblotted with Mfa1 antiserum to assess the stability of the Mfa1 polymer (Figure 3). A ladder-like polymerization pattern was observed in the *mfa3*-5 mutants at sub-boiling temperatures (60° and 80°C), demonstrating that Mfa1 polymerization occurs independently of Mfa3-5. Interestingly, neither a ladder-like pattern nor a monomeric band was observed for the *mfa2* mutant at 60°C (or at room temperature, not shown). Given that Mfa2 has been shown to regulate fimbrial length and its absence leads to longer fimbriae (Hasegawa et al., 2009), it may be the case that the Mfa1 polymer failed to enter the gel due to a longer length with potentially higher affinity among individual Mfa1 subunits. Nonetheless, a ladder-pattern was evident for the *mfa2* mutant treated at 80°C, and a monomeric band of 67 kDa was observed with all the *mfa*2-5 mutants upon sample processing at 100°C. Therefore, we concluded that Mfa1 polymerization does not require any of the accessory proteins and occurs solely by an Mfa1 subunit-mediated mechanism. Similarly, other studies have reported that loss of Mfa5 did not affect Mfa1 polymerization although the total amount of fimbrial expression was reduced (Hasegawa et al., 2016). Mfa4 has also been shown to be unnecessary for Mfa1 polymerization; however, loss of Mfa4 in a Δ*fimA* background affected thermal stability of the Mfa1 polymer (Ikai et al., 2015). We are currently investigating the reasons for a lack of difference in thermal stability of Mfa1 in the *mfa4* mutant observed here, which may relate to the presence or absence of FimA.

### Role of arginine gingipains in Mfa1 polymerization

**Figure 4:**
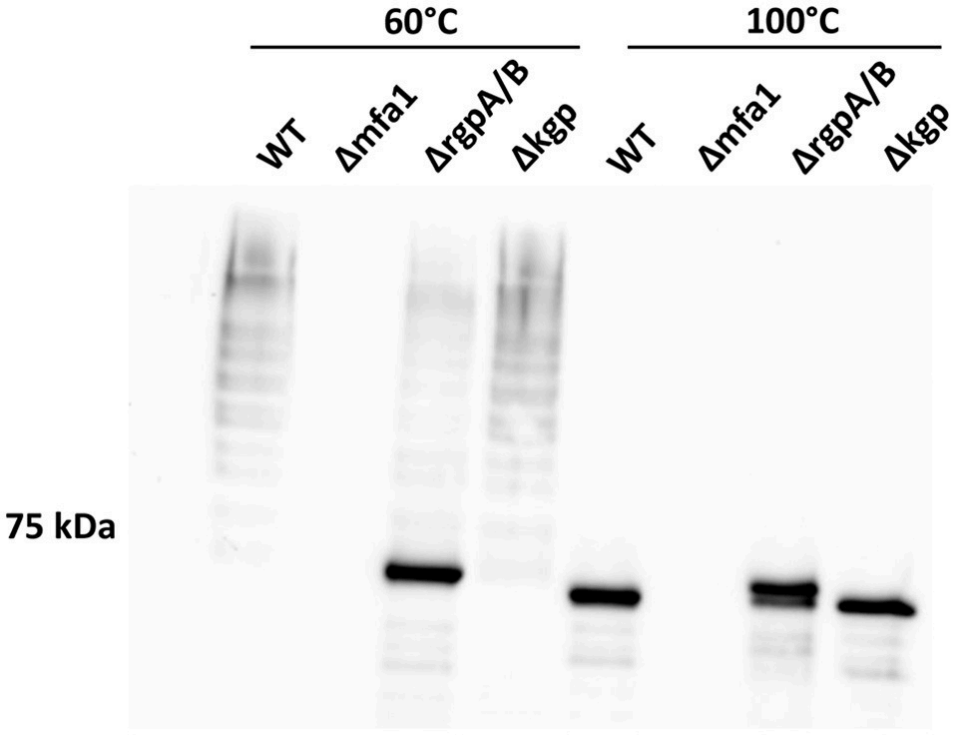
Arginine protease activity is required for Mfa1 polymerization. Whole cell lysates of 33277 WT, Δ*mfa1*, Δ*rgpA/B*, and Δ*kgp* were treated at 60° or 100°C prior to SDS-PAGE followed by immunoblotting with antibodies to Mfa1. Results are representative of 3 biological replicates.

While individual Mfa proteins can be processed by arginine gingipains (Shoji et al., 2004; Ikai et al., 2015), and the cell surface of an arginine gingipain mutant is devoid of fimbriae (Nakayama et al., 1996), the role of arginine gingipains in Mfa1 polymerization has not been established. Using a *P. gingivalis* mutant lacking both the *rgpA* and *rgpB* gingipains, we found that the arginine gingipains are necessary to observe a wild-type Mfa1 polymerization pattern following treatment of the cells at 60°C (Figure 4). A weak ladder-like pattern was observed with the Δ*rgpA/B* mutant, which may be attributed to other *P. gingivalis* proteases rescuing, albeit inefficiently, the processing of Mfa1. In contrast, loss of the lysine-gingipain, Kgp, which is not involved in Mfa1 protein processing (Kadowaki et al., 1998), had no effect on Mfa1 polymerization as no distinct monomeric band was seen at 60°C. When the bacterial cell lysates were treated at 100°C, the monomeric band for the Δ*rgpA/B* mutant appeared at a higher molecular weight compared to the wild-type and *kgp* mutant (Figure 4), demonstrating the pre-Rgp processed nature of the Mfa1 monomer. Collectively, these results suggest that proteolytic processing of Mfa1 by RgpA/B initiates the polymerization of Mfa1.

### Role of N and C terminal regions of Mfa1 in polymerization

**Figure 5:**
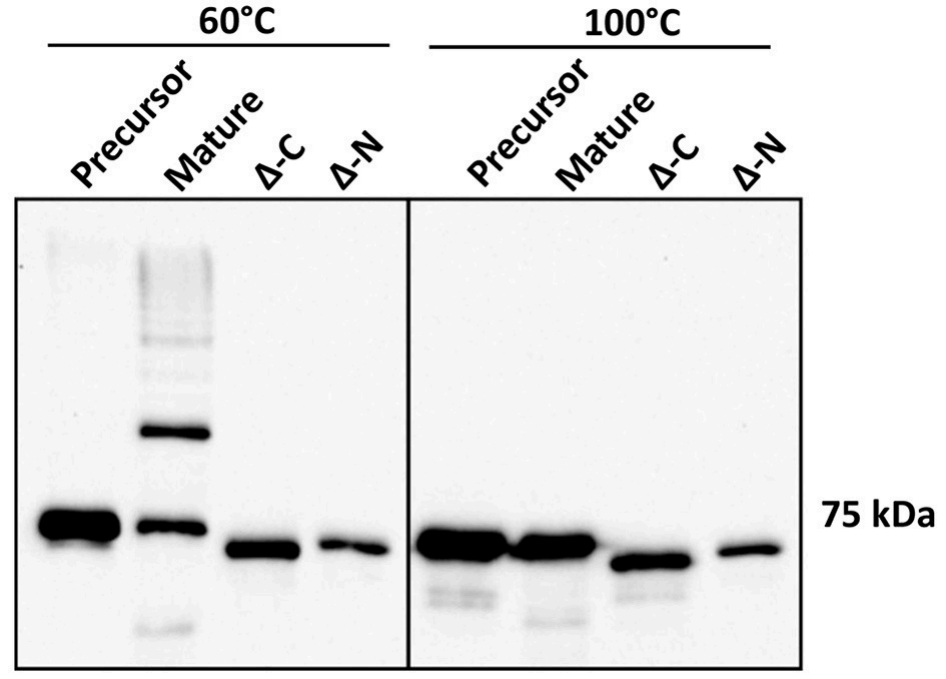
N-terminal and C-terminal deletion mutants of Mfa1 fail to polymerize. Mfa1 proteins corresponding to pre-Rgp processed form (precursor), post-Rgp processed form (mature), and either C-terminally truncated (Δ-C) or N-terminally truncated (Δ-N) forms were treated separately at either 60° or 100°C prior to SDS-PAGE and immunoblotting with antibodies to Mfa1. Results are representative of 3 biological replicates.

Structural evidence from Mfa1, Mfa4, and FimA crystals suggests DSE as a likely mechanism for polymerization of *P. gingivalis* fimbriae (Kloppsteck et al., 2016; Xu et al., 2016; Hall et al., 2018). Furthermore, truncation of a C-terminal region of Mfa1 abrogated its polymerization (Xu et al., 2016; Hall et al., 2018). Crystal structure of Mfa1 (Hall et al., 2018) reveals putative β-strand regions immediately following arginine 49, which is the Rgp processing site (Shoji et al., 2004), as well as in the C-terminus. Hence, we generated N or C-terminally truncated recombinant Mfa1 proteins such that the regions containing two consecutive β-strands that may act as donor-strands were deleted (amino acid residues 50–71 and 544–563, respectively). Recombinant Mfa1 proteins representing precursor, mature, and N or C-terminally truncated forms were examined by immunoblotting with Mfa1 antibodies. Following processing at 60°C prior to electrophoresis, monomeric bands were observed for precursor and N or C-terminally truncated Mfa1 proteins, with a characteristic ladder-like pattern only observed for mature Mfa1 protein (Figure 5). Monomeric bands only were observed when the proteins were processed at 100°C, confirming that the ladder pattern for the mature Mfa1 protein represented Mfa1 polymers. These results indicate that N and C-terminal β-strand regions are required for Mfa1 polymerization, although a truncation of either terminus could lead to a loss of tertiary structure, yielding an artificial non-native conformation that nullified the polymerization competent state.

### Role of the Mfa1 terminal β-strands in polymerization

**Figure 6:**
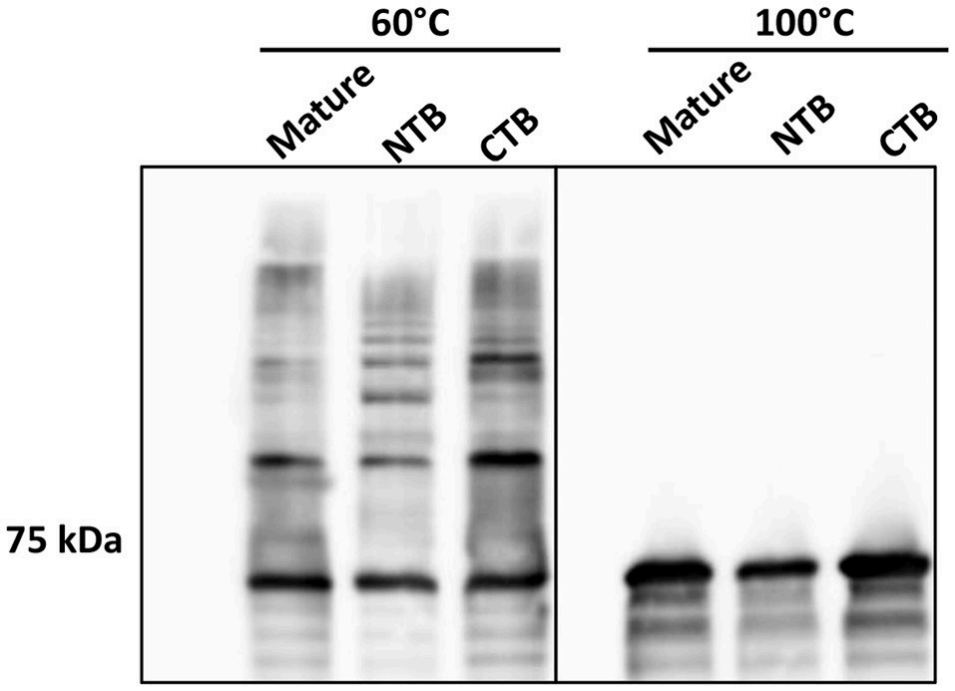
Mfa1 polymerization is unaffected by N-terminal or C-terminal β-strand disruption. Mature Mfa1 protein or derivatives with the N-terminal β-strand proline-substituted (NTB), or the C-terminal β-strand proline substituted (CTB) were treated separately at 60° or 100°C prior to SDS-PAGE and immunoblotting with antibodies to Mfa1. Results are representative of 3 biological replicates.

To further investigate the role of the β-strands located in the terminal regions of Rgp-processed Mfa1 in polymerization, two additional recombinant Mfa1 proteins were generated with mutations in the putative β-strand regions. V76 and E90 in the N-terminal region or V549 and V556 in the C-terminal region were replaced with β-strand disrupting prolines (Figure S3). As shown in Figure 6, both the N-terminal proline substituted, and the C-terminal proline substituted, Mfa1 proteins exhibited the characteristic ladder-like pattern similar to the control wild type Mfa1 at 60°C. All proteins also yielded a monomeric band at 100°C. Although disruption of β-strands was not confirmed by structural analyses, these results indicate that the polymerization of Mfa1 can tolerate substitutions with β-strand disrupting proline residues.

### Role of Mfa1 terminal hydrophobic residues in polymerization

**Figure 7:**
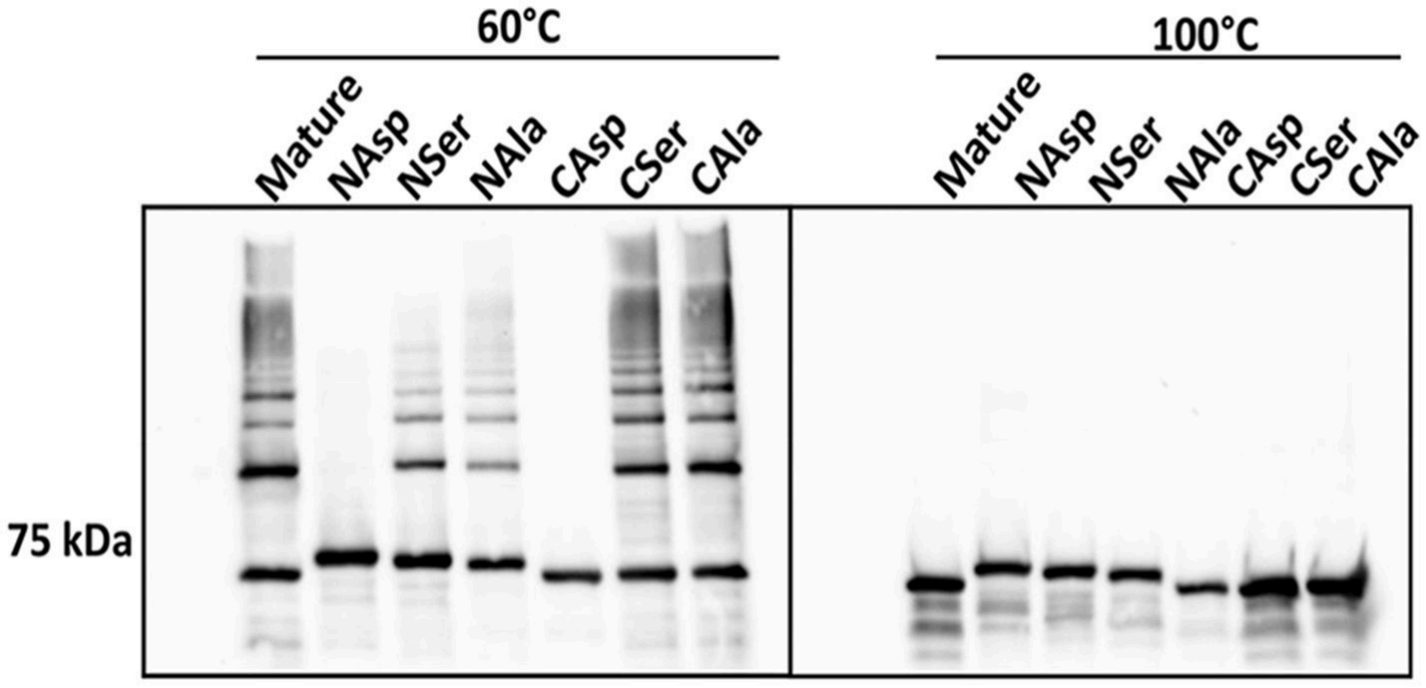
Mfa1 polymerization is prevented by substitutions in hydrophobic domains. Mature Mfa1 protein or derivatives with N-terminal or C-terminal substitutions were heated separately to 60° or 100°C prior to SDS-PAGE, and immunoblotting with antibodies to Mfa1. N-terminal (N) or C-terminal (C) aspartic acid, serine, or alanine substituted derivatives are indicated. Results are representative of 3 biological replicates.

Another feature of DSE involves alternating hydrophobic residues forming an interface between individual subunits (Waksman and Hultgren, 2009; Hospenthal et al., 2017). In silico analysis indicated that I73, V76, I78, and V81 in the N-terminus, and V547, V549, and V551 in the C-terminus are hydrophobic amino acid residues that are predicted to be buried (Figure S3). These residues were substituted with either aspartic acid, serine, or alanine, which contain a charged, a polar, or a different hydrophobic side chain, respectively, and represent a decreasing likelihood of disrupting hydrophobic interactions. In immunoblot analyses, the derivatives of Mfa1 with aspartic acid-substitutions in either terminus did not exhibit any ladder-like polymerization pattern, while the serine-substituted Mfa1 and the alanine substituted Mfa1 in either terminus showed a characteristic polymerization pattern at 60°C (Figure 7). All the Mfa1 proteins only showed monomeric bands at 100°C. The results indicate that introduction of charged residues, but not polar or different hydrophobic residues, in either N or C-terminus of Mfa1 in place of the native hydrophobic residues disrupts the polymerization of Mfa1.

**Figure 8:**
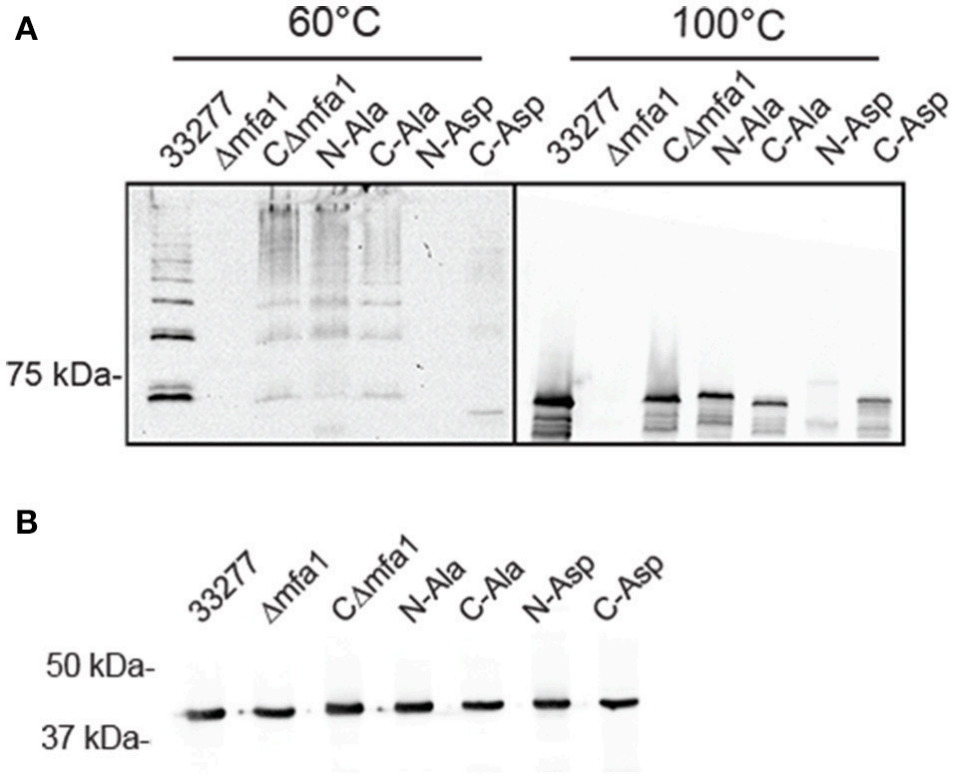
Mfa1 fails to polymerize in *P. gingivalis* strains expressing substitutions in hydrophobic domains. *P. gingivalis* strains 33277, Δ*mfa1*, or Δ*mfa1* complemented with pT-COW containing *mfa1* wild type allele or the N or C terminal alanine or aspartic acid substitutions were treated at 60° or 100°C prior to SDS-PAGE and immunoblotting with antibodies to **(A)** Mfa1 or **(B)** FimA. Results are representative of 4 biological replicates.

In order to validate the importance of the hydrophobic residues *in vivo*, genes with the corresponding site-directed mutations were cloned into the shuttle vector pT-COW and expressed from the native *mfa1* promoter in the Mfa1-deficient mutant Δ*mfa1*. RT-PCR confirmed mRNA expression in each of the recombinant strains (Figure S4). Immunoblot analysis (Figure 8A) showed that Mfa1 in cell lysates of strains with either the N-terminal or C-terminal aspartic acid substitutions failed to polymerize. Furthermore, not only did Mfa1 with the aspartic acid substitutions fail to polymerize, monomeric Mfa1 was either reduced (C-terminal) or absent (N-terminal) after heating the lysates to 100°C. In contrast, N-terminal or C-terminal alanine substitutions were tolerated, and Mfa1 polymerization occurred. One possible explanation for the lower levels of Mfa1 with aspartic acid substitutions is that misfolded Mfa1 monomeric subunits accumulate in the periplasm and activate a degradation pathway. This could potentially involve PGN_0637 (HtrA) which is annotated as a serine protease and has around 37% homology to the DegP periplasmic protease of *E. coli*, known to degrade accumulating proteins in the periplasm (Waksman and Hultgren, 2009). Immunoblot analysis of the Mfa1 substituted strains with antibodies to the FimA fimbriae showed no change in expression levels (Figure 8B), supporting the notion that misfolded Mfa1 is targeted for degradation. Collectively, these results underscore the essential aspect of the alternating hydrophobic residues in either terminus in the polymerization of Mfa1, supporting a DSE-like process as the Mfa1 polymerization mechanism.

## Discussion

The FimA and Mfa1 fimbrial structures of *P. gingivalis* make important contributions to attachment, colonization and virulence (Lamont and Jenkinson, 1998; Enersen et al., 2013). Distinct features in *P. gingivalis* such as the absence of chaperones and ushers, along with the two-step maturation process and lack of homology with fimbriae in other organisms, have led to the categorization of FimA and Mfa1, along with related structures in the Bacteroidia, as Type V pili (Xu et al., 2016; Hospenthal et al., 2017). Both FimA and Mfa1 contain accessory proteins which assemble on the fimbrial tip (Pierce et al., 2009; Hasegawa et al., 2013, 2016; Ikai et al., 2015). However, little is known regarding interactions among the Mfa3-5 accessory proteins or their role, if any, in Mfa1 polymerization. In this study, using an *in vitro* assay we found that Mfa3 can bind to Mfa1 along with Mfa4 and Mfa5, whereas Mfa4 and Mfa5 do not bind to each other or to Mfa1. Thus, assembly of the tip complex would appear to depend on the promiscuous binding of Mfa3 which links the other Mfa proteins together. Previous studies have shown that the absence of any one of the Mfa3, Mfa4, or Mfa5 proteins results in loss of the tip complex (Ikai et al., 2015; Hasegawa et al., 2016), indicating that once initiated by Mfa3, stability of the tip complex is enhanced through the multi-subunit interactions. Indeed, it has been proposed that the N-terminus of mature Mfa3 can displace the β1a strand of Mfa4, and allow polymeric assembly of the tip complex (Kloppsteck et al., 2016). Mfa2, which can bind to Mfa1 and terminate fimbrial elongation, also bound to Mfa3 and Mfa5, suggesting a role for Mfa2 in regulating the development of the tip complex. Additionally, the association of Mfa3 and Mfa5 with Mfa2 may represent a mechanism by which *P. gingivalis* terminates its fimbrial assembly. Binding to Mfa3 and Mfa5 (which themselves are bound to each other) instead of Mfa1, could prevent further assembly by blocking Mfa3/Mfa5 association with Mfa1. The formation of the Mfa1 polymer was independent of formation of the tip complex, as Mfa1 polymerization was observed in each of mutants lacking Mfa3, Mfa4 or Mfa5, consistent with previous studies (Hasegawa et al., 2013, 2016; Ikai et al., 2015).

The RgpA and RgpB gingipain proteases are recognized as important virulence factors of *P. gingivalis* with potent activity against host cell matrix proteins and immune effector molecules (Lamont and Jenkinson, 1998; Guo et al., 2010; Li and Collyer, 2011). In addition to action on host substrate, Rgps also play a role in post-translational processing of *P. gingivalis* proteins. RgpA and Kgp, along with the HagA hemagglutinin protein, contain hemagglutinin domains which are released from the pro-proteins by gingipain cleavage and assemble into multifunctional, multidomain adhesive complexes on the bacterial surface (Guo et al., 2010). The FimA fimbrial subunit FimA is secreted as a precursor that requires Rgp cleavage to remove the diacylglyceride-modified N-terminus and allow membrane anchoring and polymerization (Guo et al., 2010). Structural evidence suggests that prior to processing by an Rgp arginine gingipain protease, the C-terminal β-strands of FimA remain folded close to the protein core, while the N-terminal β-strands cover the hydrophobic groove necessary for DSE (Xu et al., 2016). Similarly, through examining the crystal structure of Mfa4, it has been proposed (Kloppsteck et al., 2016) that the N-terminal β-strands prior to the Rgp cleavage site may serve as an intrinsic chaperone by improving the solubility and stability of Mfa4, likely through covering the hydrophobic groove by the N-terminal β-strands. An RgpA/B deficient mutant of *P. gingivalis* is devoid of Mfa1 fimbriae (Nakayama et al., 1996), and hence Rgp activity has been proposed as crucial for Mfa1 fimbrial production. In this study we provide the first experimental evidence that this is the case, and that in the absence of RgpA and RgpB, Mfa1 polymerization does not occur. Hence both the Mfa1 and FimA fimbrial structures appear to be processed through a similar, if not identical, assembly mechanism.

Donor strand exchange (DSE) is essential for the biogenesis of the chaperone-usher mediated fimbrial/pili system including type 1/P pili of *E. coli* (Remaut et al., 2006; Proft and Baker, 2009; Waksman and Hultgren, 2009; Zav'yalov et al., 2010), and has been proposed as the mechanism of polymerization for both the FimA and Mfa1 fimbriae despite the apparent absence of ushers or fimbrial chaperones in *P. gingivalis* (Kloppsteck et al., 2016; Xu et al., 2016). In the case of FimA, the N-terminal β-strand occupies the NTD groove and thus functions as an isogenous chaperone. The NTD groove is subsequently uncovered by Rgp processing and becomes available for interaction with a C-terminal β-strand on an adjacent subunit (Xu et al., 2016). In Mfa1, structural and functional studies have not definitively established either the presence of an endogenous chaperone or the identity of the donor strand (Hall et al., 2018). The results here show that both the N- and C- terminal regions of Mfa1 are necessary for polymerization. Proline substitution in the N- or C- terminal β-strand regions has no effect on polymerization indicating either a failure to significantly disrupt the hydrogen bonding pattern between β-strands or that the β-strands *per se* are not essential for DSE. In Mfa1, the terminal β-strands may not be essential for completion of an intrinsic structural fold, and it is possible that other secondary structural elements or hydrophobic domains may participate in completing the necessary structure. Indeed, another feature of DSE in *E. coli* pili is the involvement of hydrophobic residues (Waksman and Hultgren, 2009). The hydrophobic C-terminal groove which runs along the length of the pilin contains five hydrophobic pockets which can interact with hydrophobic residues on the donor strand (Hospenthal et al., 2017). *In silico* analysis of Mfa1 showed there are several alternating amino acid residues in both N and C termini that are predicted to have hydrophobic properties. When these amino acids were substituted with aspartic acid, polymerization of Mfa1 was disrupted. In contrast, Mfa1 polymerization was able to proceed in the presence of serine or alanine substitutions. As substitutions with serine, which possesses polar side-chains, did not disrupt the polymerization of Mfa1, the results also indicate that the hydrophobicity in the binding interface can tolerate some degree of polarity, and the polymerization interface surrounding the alternating hydrophobic residues in either terminus of Rgp-processed Mfa1 likely does not involve a strict hydrophobic milieu but rather a state intermediate between strict hydrophobicity and strict hydrophilicity. These results thus provide novel insights into the nature of the polymerization mechanism of the Type V pili of *P. gingivalis*.

## Author contributions

JL, LW, CC, and DM conceived and performed the experiments. RL, and YH conceived overall plan, and interpreted data. All authors wrote sections of the manuscript.

### Conflict of interest statement

The authors declare that the research was conducted in the absence of any commercial or financial relationships that could be construed as a potential conflict of interest.
