# Supplementary material for: Maturation of the Mfa1 Fimbriae in the Oral Pathogen Porphyromonas gingivalis
Source: Front Cell Infect Microbiol. 2018 May 9;8:137. doi: 10.3389/fcimb.2018.00137 (PMC5954841; doi:10.3389/fcimb.2018.00137)
Supplement: Supplementary file 1 [file Presentation_1.PDF]

A

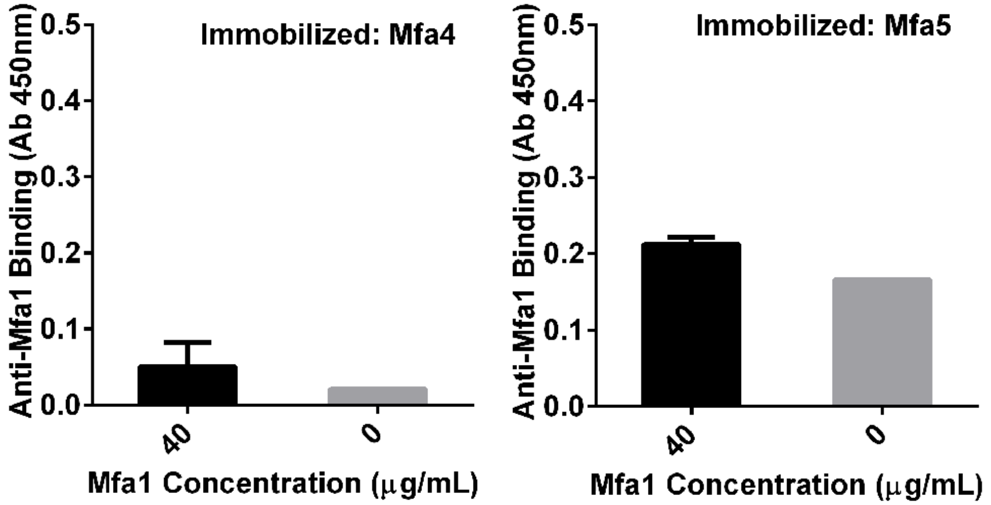

B

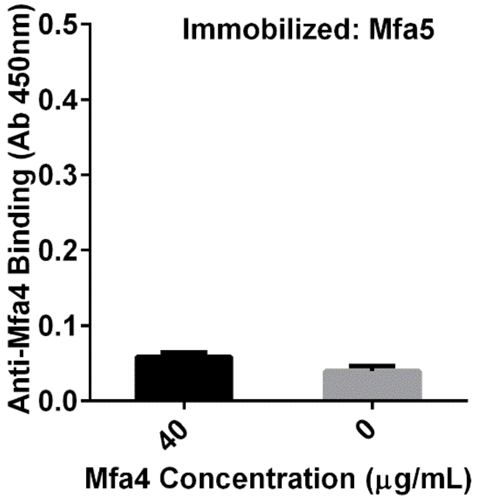

Figure S1. ELISA of binding among Mfa proteins. A. Rgp-processed Mfa4, or recombinant Mfa5 (1 μg) were immobilized on a microtiter plate. Binding of Rgp-processed Mfa1 at the concentrations indicated was detected with antibodies to Mfa1 (1:5000). B. Recombinant Mfa5 (1 μg) was immobilized on a microtiter plate and binding of Rgp-processed Mfa4 at the concentrations indicated was detected with antibodies to Mfa4 (1:5000). In both A and B, reactions were developed with secondary anti-rabbit IgG HRP-linked antibodies (1:5000) and TMB substrate. Error bars are SD (n=3). One representative result of three independent experiments is shown. Values were not significantly different by ANOVA.

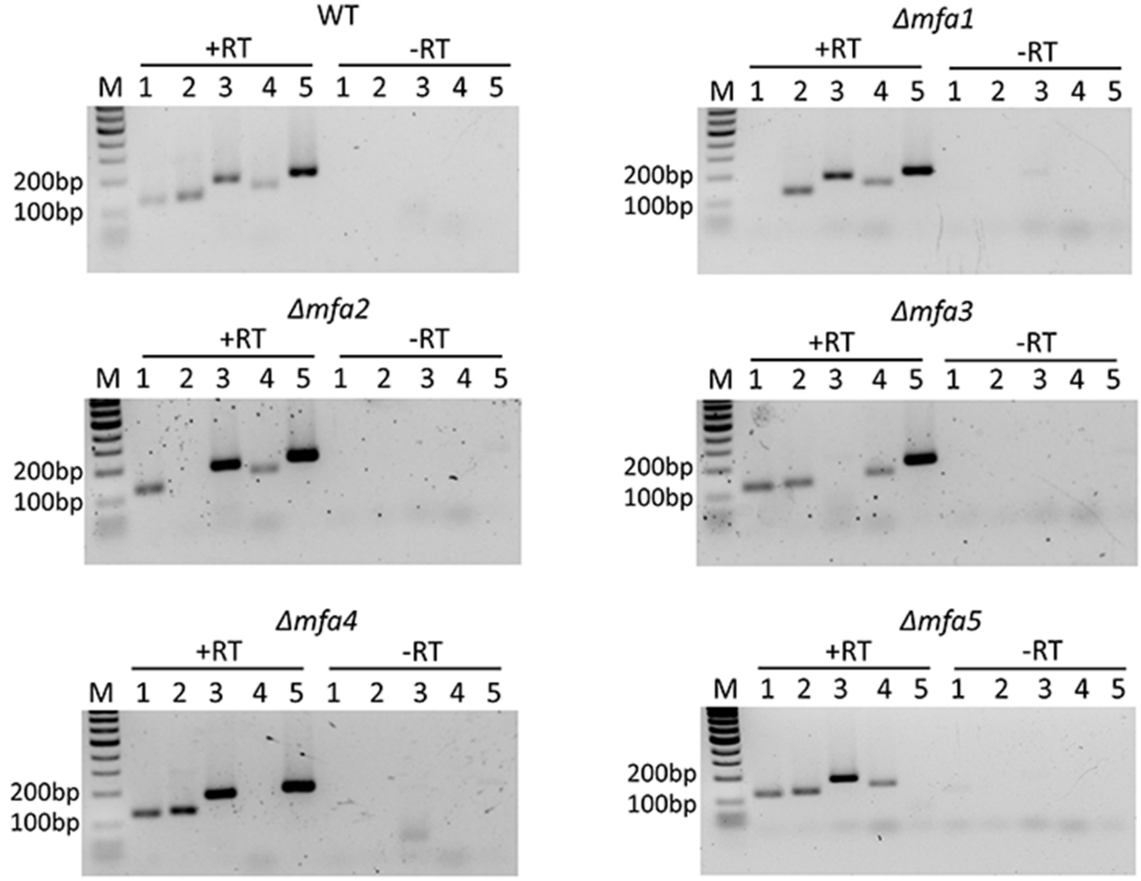

Figure S2. Transcriptional analysis of *mfa* gene cluster in *mfa* mutants. Total RNA was extracted from *P. gingivalis* 33277 WT, Δ*mfa1*, Δ*mfa2*, Δ*mfa3*, Δ*mfa4*, and Δ*mfa5* as indicated and used for RT-PCR of the *mfa* gene cluster. Numbers above lane represent the *mfa* gene from which the primers are derived. A control with no reverse transcriptase (-RT) was included. Results are representative of 3 biological replicates

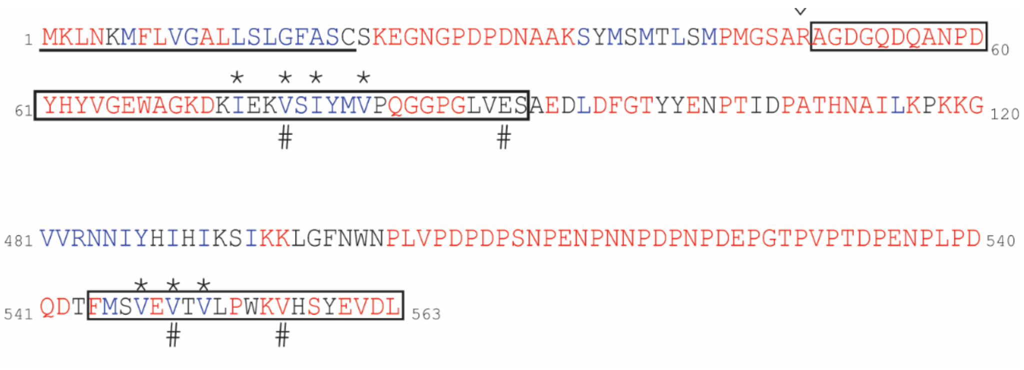

Figure S3. Schematic of predicted Mfa1 biochemical properties and mutations. The N-terminal (upper) and C-terminal (lower) amino acid sequences with biochemical properties generated using RaptorX Property Prediction. Residues that are predicted to non-polar and surface exposed are colored in red, while residues that are predicted to be hydrophobic and buried within the structure are colored in blue. Residues 1-20 are underlined to highlight the signal sequence. Arg49 is the site of gingipain cleavage and is shown with an arrowhead. The regions of sequence that were truncated for the N- and C-terminal truncations are shown in the black boxes. In order to break the N- and C-terminal β-strands; V76 and E90, or V549 and V556 were replaced with proline (substitutions indicated with #). The amino acids substituted in order to replace hydrophobic amino acids within the N- and C-terminal β-strands are indicated with *.

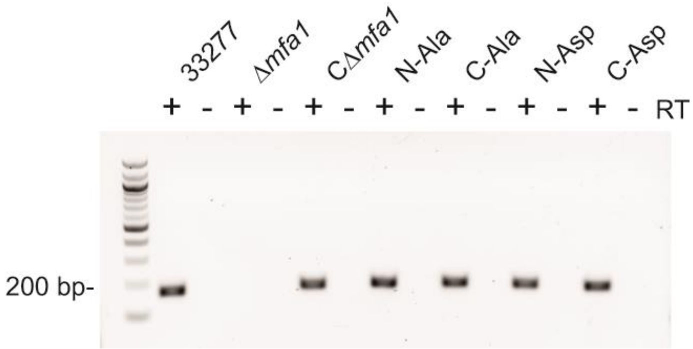

Figure S4. Transcriptional analysis of *mfa1* in strains with substitutions in Mfa1 hydrophobic domains. RT-PCR of *mfa1*. Total RNA was extracted from *P. gingivalis* strains 33277 WT, Δ*mfa1,* Δ*mfa1* complemented with pT-COW containing *mfa1* wild type allele (CΔ*mfa1*) or the N or C terminal alanine (Ala) or aspartic acid (Asp) substitutions. Size markers are on the left side. A control with no reverse transcriptase (-RT) was included. Results are representative of 3 biological replicates.
