# Supplementary material for: Maturation of the Mfa1 Fimbriae in the Oral Pathogen Porphyromonas gingivalis
Source: Front Cell Infect Microbiol. 2018 May 9;8:137. doi: 10.3389/fcimb.2018.00137 (PMC5954841; doi:10.3389/fcimb.2018.00137)
Supplement: Supplementary file 2 [file Table_1.DOCX]

Table S1. Primers used in this study

| Name^1^ | Primer sequence (5’ – 3’)^2^ | Function |
| --- | --- | --- |
| 1N50F | GATGATGATGAT**AAA**GCGGGTGACGGACAGGAT | For expression of Mfa1 and Mfa1 with N-terminal deletion |
| 1N563R | TTAGAGATCAACCTCATAGGAATGAAC |  |
| 1NS287F | CACCAGTAAAGAGGGCAATGGCC | For expression of precursor Mfa1 |
| 1NS287R | TTAGAGATCAACCTCATAGGAATGAAC |  |
| 2N29F | GATGATGATGATAAATGTGATAAGATGATTTATGACAATTACG | For expression of Mfa2 |
| 2N324R | TTAAAGTTCTATTTCGTAACTATGTATCAACC |  |
| 3N44F | GATGATGATGAT**AAA**GCAGCACATACGAATGGC | For expression of Mfa3 |
| 3N446R | CTATTTCTTGATAAAAACTTTATCCGG |  |
| 4NS290F | CACCAAGAACAATCCTAGCGAGCC | For expression of Mfa4 |
| 4NS290R | TCAAATCTCGACTTCGTACTTGTAC |  |
| 291NTF | CACCTTTCAAATAAAAGCTCGCCCT | For expression of Mfa5 with C-terminal deletion |
| 291NTR | TTAGTCGAATCCGAACGAAAG |  |
| 1R543 | TTACGTATCCTGATCAGGCAAGG | For expression of Mfa1 with N-terminal deletion |
| 1F92 | GATGATGATGAT**AAA**GCTGAAGATCTTGATTTTGGC | For expression of Mfa1 with C- terminal deletion |
| MFA1F | GCTTGTGGAGAGTGCTGAAG | For qRT-PCR |
| MFA1R | TTGCCGACAGCAGAATTAAC |  |
| MFA2F | ATAGATGGGACGACCCTTTG |  |
| MFA2R | ACACTCACCGTCACACGATT |  |
| MFA3F | TGGCCTCGATCGTGAACAAA |  |
| MFA3R | ATTGTTTTCTCCGTCCGGCT |  |
| MFA4F | TGCTGCCGAAAGGCTCATTA |  |
| MFA4R | CCAGCCTCGGATTGTGTCAT |  |
| MFA5F | GGCTTCGATGCGGATAAGGA |  |
| MFA5R | CTGCCGATTCAACCCACTCT |  |
| M1NHF | CGATTACATGGATCCTCAGGGTGGCCCTGGG | For placement of aspartic acids in N-terminal Mfa1 |
| M1NHR | CTATCTTTCTCATCTTTGTCTTTTCCTGCCCACTCTCCTAC |  |
| M1NSF | CTCTTACATGTCTCCTCAGGGTGGCCCTGGG | For placement of serines in N-terminal Mfa1 |
| M1NSR | CTAGATTTCTCAGATTTGTCTTTTCCTGCCCACTCTCCTAC |  |
| M1NAF | CGCTTACATGGCTCCTCAGGGTGGCCCTGGG | For placement of alanines in N-terminal Mfa1 |
| M1NAR | CTAGCTTTCTCAGCTTTGTCTTTTCCTGCCCACTCTCCTAC |  |
| M1CHF | TACAGATTTGCCTTGGAAAGTTCATTC | For placement of aspartic acids in C-terminal Mfa1 |
| M1CHR | TCCTCATCCGACATGAACGTATCCTG |  |
| M1CSF | TACATCTTTGCCTTGGAAAGTTCATTC | For placement of serines in C-terminal Mfa1 |
| M1CSR | GACTCAGACGACATGAACGTATCCTG |  |
| M1CAF | TACAGCTTTGCCTTGGAAAGTTCATTC | For placement of alanines in C-terminal Mfa1 |
| M1CAR | GCCTCAGCCGACATGAACGTATCCTG |  |
| M1NBF | GGGTGGCCCTGGGCTTGTGCCAAGTGCTGAAGATCTTGATTTTG | For placement of prolines in N-terminal Mfa1 |
| M1NBR | TGAGGCACCATGTAGATGCTTGGTTTCTCAATTTTGTCTTTTCC |  |
| M1CBF | CCTTGGAAACCACATTCCTATGAGGTTGATCTC | For placement of prolines in C-terminal Mfa1 |
| M1CBR | CAAAACTGTTGGCTCAACCGACATGAACGTATC |  |
| RGPAUS_F | GTTCAGATTGTCCGGCTGGAGAATAGGCAGAC | Generating fragment upstream of *rgpA* for use with allelic exchange mutation |
| RGPAUStet_R | AGCATTAGAACTTGGCAATAAATTCTGTCTTGGACTCGGAGAC |  |
| RGPADStet_F | CTACGTTAAGGAGATAATTCGTTGTGTTTTTCATTTTGATGAAATTAG | Generating fragment downstream of *rgpA* for use with allelic exchange mutation |
| RGPADS_R | GTCAGAAAAAGCCTTCCGAATCCGACAAAGATAG |  |
| RGPB US_F | CTGCCTTTCTATCTGGCCATGTGGATGTGCTAC | Generating fragment upstream of *rgpB* for use with allelic exchange mutation |
| RGPBUSerm_R | GATGGAGCGGAAACGTAAAAGATTCACACTGCAATTCTCTAATAAG |  |
| RGPBDSerm_F | ACGGGCAATTTCTTTTTTGTCATTTGCTTGAATTAGTTTTTTATTTG | Generating fragment downstream of *rgpB* for use with allelic exchange mutation |
| RGPBDS_R | GAAACCCGAATGGTTGAAAATACGTCTTGGTGGGAATGAG |  |
| KGPUS_F | CACATTTCGGTAAGGGAAGGGGTGCTTGTGGATG | Generating fragment upstream of *kgp* for use with allelic exchange mutation |
| KGPUSerm_R | TGGAGCGGAAACGTAAAAGATTCTGTCTTGGACTCGGAG |  |
| KGPDSerm_F | ACGGGCAATTTCTTTTTTGTCATACTTTAAAACAATTTATGGTC | Generating fragment downstream of *kgp* for use with allelic exchange mutation |
| KGPDS_R | CAGCCGAGGAGCATACGGATATTCGCTTG |  |
| MFA1usF | CTCAATGTAAAAGGAGAAAAGAAGGTAAGAAGGCTATG | Generating fragment upstream of *mfa1* for use with allelic exchange mutation |
| MFA1usR | ACGGGCAATTTCTTTTTTGTCATAAGCCAAATGTTTAAAAG |  |
| MFA1dsF | GATGGAGCGGAAACGTAAAAGATTAGCTATTGTAAAATTTTC | Generating fragment downstream of *mfa1* for use with allelic exchange mutation |
| MFA1dsR | CACATCATTGCAACTGCCCTGACGATACTTATG |  |
| MFA2usF | CAACGAAAGCCCAGAGTTATGAAATTAAAGCCAC | Generating fragment upstream of *mfa2* for use with allelic exchange mutation |
| MFA2usR | GGCAATTTCTTTTTTGTCATTGTTTTAAAAAATATAGAGGGTG |  |
| MFA2dsF | TGGAGCGGAAACGTAAAAGA GAGAAAAAAGACCGGTTCTTC | Generating fragment downstream of *mfa2* for use with allelic exchange mutation |
| MFA2dsR | CATTGGTTCGTGGCACCTACTCGCCTCAG |  |
| MFA3usF | GCAGTGCCAA TGTGTTTGAGGATGTCCAGTTG | Generating fragment upstream of *mfa3* for use with allelic exchange mutation |
| MFA3usR | GGCAATTTCTTTTTTGTCATATTCCAAGTGTATATGGTTATAAG |  |
| MFA3dsF | TGGAGCGGAAACGTAAAAGA ACAGACTTATGAAAAAGTATTTG | Generating fragment downstream of *mfa3* for use with allelic exchange mutation |
| MFA3dsR | GTCCGGCAAAGGCAAGCGATAAAGCTC |  |
| MFA4usF | CTACCTATGTCTTTACTGTGAAATTGAAACCCGGAC | Generating fragment upstream of *mfa4* for use with allelic exchange mutation |
| MFA4usR | GGCAATTTCTTTTTTGTCATAAGTCTGTCTATTTCTTG |  |
| MFA4dsF | TGGAGCGGAAACGTAAAAGATATTTTTTAGGTCTTGTTTGATATTG | Generating fragment downstream of *mfa4* for use with allelic exchange mutation |
| MFA4dsR | GAGTTGGATAATTTGGAGTAAGAGGTGTAGATGGATTG |  |
| MFA5usF | GTGAGACAGTACGCTATGAAAGGAATCAAGGAAG | Generating fragment upstream of *mfa5* for use with allelic exchange mutation |
| MFA5usR | GGCAATTTCTTTTTTGTCATTATTATTACCTCGTTAGTTACTACC |  |
| MFA5dsF | TGGAGCGGAAACGTAAAAGATAATGTGAAGAAGATGATGC | Generating fragment downstream of *mfa5* for use with allelic exchange mutation |

^1^ F: forward; R: reverse ^2^ Enterokinase P1 site is bolded and underlined.
